# Effects of green and red light in β_L_-crystallin and ovalbumin

**DOI:** 10.1038/srep18120

**Published:** 2015-12-14

**Authors:** J. Horacio Espinoza, Elizabeth Reynaga-Hernández, Jaime Ruiz-García, Gabriela Montero-Morán, Margarita Sanchez-Dominguez, Hilda Mercado-Uribe

**Affiliations:** 1 CINVESTAV-Monterrey, PIIT, Nuevo León, 66600, México; 2 Instituto de Física, Universidad Autónoma de San Luis Potosí, Álvaro Obregón 64, San Luis Potosí, SLP, 78000, México; 3 Centro de Investigación en Materiales Avanzados (CIMAV, S.C.), Unidad Monterrey, Alianza Norte 202, Nuevo León, 66600, México

## Abstract

The effects of visible light on biological systems have been widely studied. In particular, the alterations of blue light on the ocular lens have recently attracted much attention. Here, we present a study about the effects produced by green and red light on two different proteins: β_L_-crystallin and ovalbumin. Based on differential scanning calorimetry (DSC), circular dichroism (CD), dynamic light scattering (DLS), and fluorescence emission measurements, we found that both wavelengths induce structural changes in these proteins. We also observed that β_L_-crystallin aggregates. Our work may advance our understanding about conformational and aggregation processes in proteins subjected to visible radiation and the possible relationship with cataracts. While blue light has been considered the only harmful component in the visible espectrum, our findings show the possibility that lower energy components may be also of some concern.

The effects produced by visible light (an electromagnetic radiation with wavelengths between 380–750 nm) on biological systems have been investigated for several years^1^,^2^,^3^,^4^,^5^,^6^,^7^,^8^,^9^. Very recently, Y. Kuse *et al.*^10^ reported a severe damage caused by blue light (from emitting diodes, LEDs) in murine cone photoreceptor-derived cells. This study may have a potential warning for humans, because we spend more and more time in front of screens that emit a large amount of blue light. M. Hori *et al.*^11^ reported, also recently, that such visible radiation kills eggs, larvae, pupae, and adults of *Drosophila melanogaster.* The authors claim that blue light is even more harmful for insects than UV radiation. Furthermore, other authors have found that overexposure to blue light may have detrimental effects on human corneal epithelial and retinal photoreceptors cells compared to other visible light wavelengths^12^,^13^.

Even lower energies of visible light (red and green) have consequences in the physiology, growth and reproduction of some organisms^3^,^4^,^6^. For instance, exposure to green light causes reduction in the radial expansion of fungi, growth of HeLa cells, retinal DNA breaks in rats, hypersensitivity of a gene in transgenic tobacco, modulated changes in the activity of collagenase enzyme and enhancement of antioxidant activity of barley^5^,^7^,^14^,^15^,^16^. Red light from monochromatic low-intensity sources can stimulate plant development, affect the expression level of oxidative stress enzymes of *E. coli* in seawater and DNA synthesis in HeLa cells, as well as the growth of other types of bacteria, yeast and different species^17^,^18^,^19^.

All in all, it has been found that the mechanisms responsible for some disturbances by specific wavelengths show the following outcomes: an increase in the reactive oxygen species (ROS), epithelial cell injury, double stranded DNA breaks (“ladder” of base pair fragments), activation of photoreceptor specific cell damage, and modifications of protein expression levels^10^,^11^,^12^,^13^,^15^.

In this context, our main concern is to understand how the above effects can take place considering the fact proteins have null absorbance at visible light wavelengths^20^. This is relevant because proteins are plenty in cells and any disturbance in them has considerable implications in the cell functioning and health of organs. For instance, it has been shown that long-term cronic exposure to blue light is one of the risk factors for development of age-related macular degeneration (AMD). Różanowska & Sarna confirmed that exposure to light accelerates the photoreceptors damage and speculated that rhodopsin is the essential protein involved in the injury of the retina^8^. In addition, it has been reported that UV light induces covalent scission of the Trps indole ring and aggregation of crystallin proteins, which are factors in the etiology of cataract formation^21^,^22^.

In the present work, we explore the effects of green and red light on two different proteins: β_L_-crystallin and ovalbumin. β_L_-crystallin is a complex protein of mostly dimers. It constitutes one of the three principal proteins in the human lens and takes part in the focusing of light on the retina, keeping the transparency and refractive characteristics of the lens. It has been suggested that one function of this protein is to maintain the solubility of other β-crystallins that are strongly modified during aging^23^,^24^,^25^,^26^,^27^. According to its size, pH, ionic strength and concentration in the lens, there are, in fact, three different β crystallin complexes: β_Low1_ (β_L1_), β_Low2_ (β_L2_), and β_High_ (β_H_). Such parameters also determine their stability. Under equilibrium conditions, β-crystallin can unfold to a bimodal transition with a stable intermediate or a state without intermediate^23^,^24^. These proteins self-associate mainly into dimers (β_L2_), tetramers (β_L1_) and high-order oligomers (β_H_) which can undergo fast subunit interchange. β_L_-crystallin exhibits N-terminal extensions or arms and it is composed of two domains connected by a short linker; the N-terminal domain of each monomer interacts with the C-terminal domain of the other. Each domain consists of two similar polypeptide chains folded to give a ’Greek key’. The core of each domain is extremely hydrophobic with a high amount of aromatic residues^21^,^23^,^28^,^29^.

Ovalbumin is the main protein of chicken egg white and the first crystallographic model for a native protein. It is a member of the serpin super-family, however, it has not any protease inhibitory property, and its function is still unknown^30^. Unlike the β_L_-crystallin this protein lacks a N-terminal leader sequence. Ovalbumin has four domains, three of them contain alpha-helix and beta strands, and the fourth is mostly alpha-helix. This protein shows a metastable folding intermediate, which is not the most thermodynamic favoured conformation^30^,^31^.

We exposed these two globular proteins to different time intervals of red and green light. We analyzed the effects produced by the radiation, measuring the changes in the calorimetric response, particle size distribution, secondary structure, and fluorescence emission. Although both proteins show an increase in enthalpy due to the radiation, indicating that cohesion increases, aggregation is observed only in β_L_-crystallin.

## Results

### Enthalpy change of irradiated proteins.

**Figure 1:**
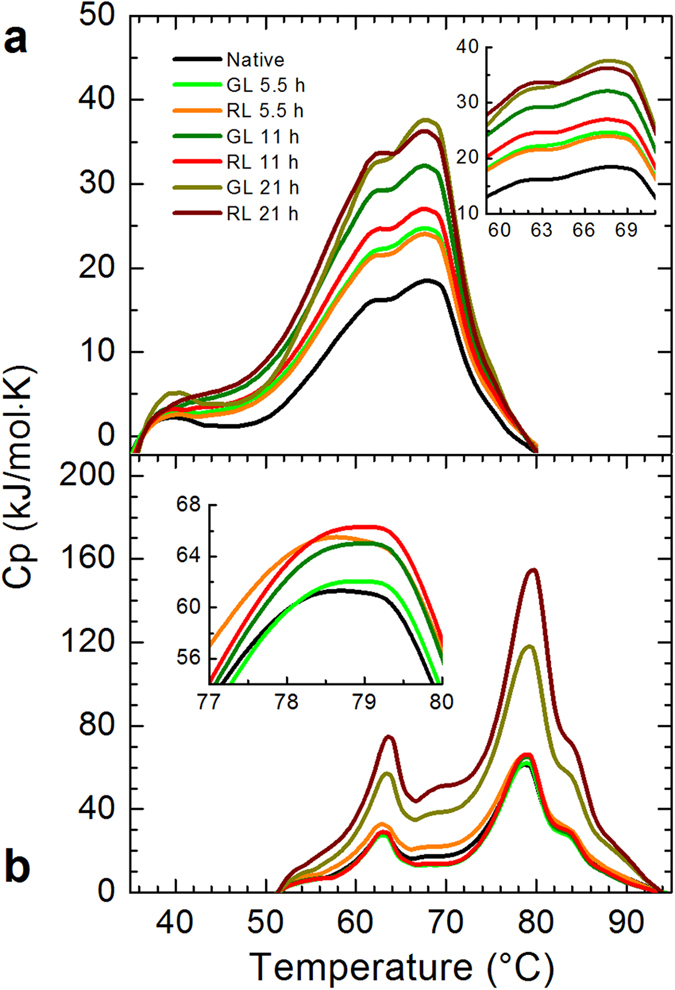
Calorimetric profiles of β_L_-crystallin and ovalbumin exposed to red and green light. β_L_-crystallin (**a**) and ovalbumin (**b**) were exposed to different time intervals (5.5, 11 and 21 h) to red and green light. Then, the samples were stored at 4 °C during 24 h and evaluated the effect of such exposures by calorimetry. The native sample (black lines) showed the main transition temperature at 67.87 ± 0.48 °C and 78.66 ± 0.02 °C for β_L_-crystallin (**a**) and ovalbumin (**b**), respectively. It is shown that both types of light affect the proteins. In the first case, the enthalpy changes are more notorious with green light, GL (green lines); in the second case is the red light, RL (red lines) that produces a more prominent change. These changes increase as the exposure time increases. For clarity, we show in the insets zooms of the two regions of the spectra.

Differential scanning calorimetry (DSC) has been acknowledged as a highly sensitive technique to study thermally induced protein folding and unfolding transitions. With this technique, conformational changes in proteins are thermodynamically characterized. To investigate about these changes in β_L_-crystallin and ovalbumin after exposure to red and green light, we evaluated the calorimetric profiles of such native and irradiated proteins (Fig. 1). The position of the calorimetric peaks is similar to those reported in the literature with different buffers^32^,^33^. However, it is evident that the area under the curve (H) increases as the irradiation time increases (from 305 to 732 kJ/mol for β_L_-crystallin (a), and from 867 to 2,252 kJ/mol for ovalbumin (b)). Both wavelengths have a notorious calorimetric effect on β_L_-crystallin (Fig. 1a), which is more pronounced with the green light, especially at intermediate times. In contrast, red light causes a major effect on ovalbumin (Fig. 1b) with a significant response at the largest exposure time (see the insets). These results illustrate that both types of light induce changes in these proteins, with the consequent increase in enthalpy. We will discuss this point in detail at the Discussion section.

### Particle size distribution analysis.

**Figure 2:**
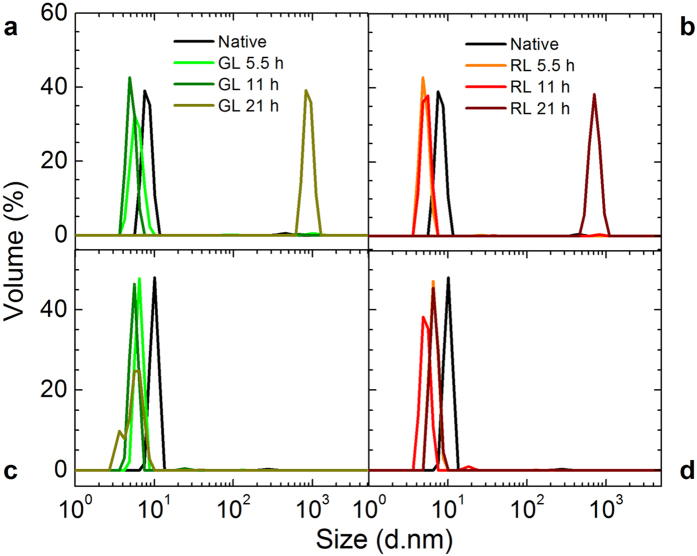
Particle size distribution of β_L_-crystallin and ovalbumin exposed to red and green light. β_L_-crystallin (**a,b**) and ovalbumin (**c,d**) were exposed to red and green light to different time intervals (5.5, 11 and 21 h) and particle distributions were evaluated. Both native proteins (black lines) exhibited average particle sizes in the nanometer range. Note the shrinkage on both proteins and formation of aggregates in β_L_-crystallin.

In order to determine the nature of the conformational changes of the proteins by exposure to red and green light, the particle size distribution was measured in terms of dynamic light scattering (DLS). Figure 2 shows how the particle size distribution of β_L_-crystallin (a–b) and ovalbumin (c–d) changes upon this radiation. First, we observe that β_L_-crystallin, which self-associates mainly into dimmers, reduces its size after exposure, indicating that light promotes more packing. But the most important effect is a significant aggregation at the highest dose. While the second protein seems to shrink also, exposure to red and green light does not produce aggregation.

### Analysis of β_L_-crystallin and ovalbumin secondary structures.

**Figure 3:**
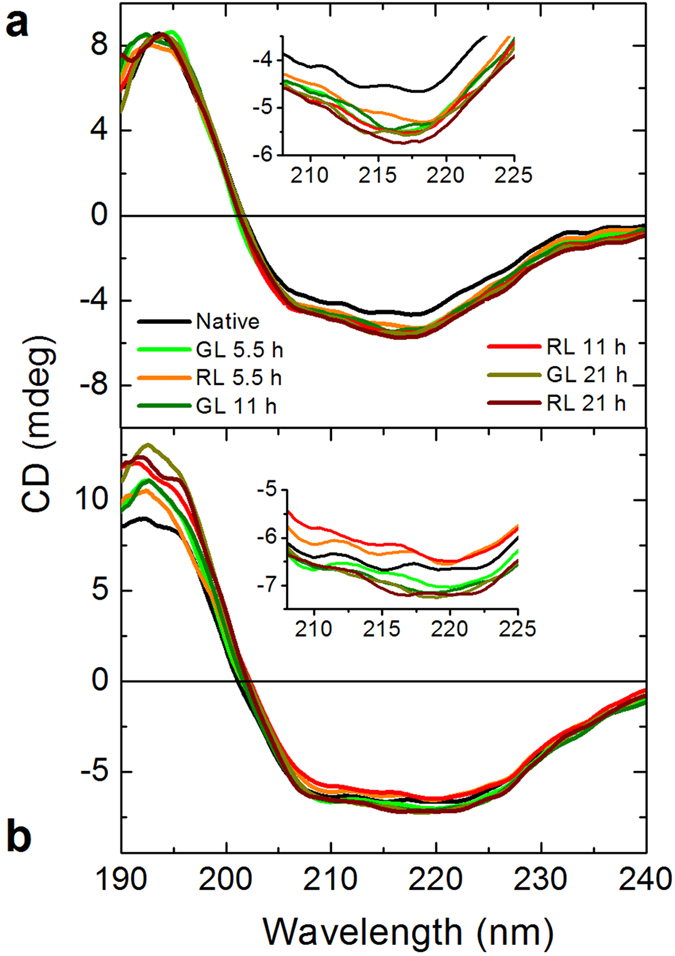
Circular dichroism study. β_L_-crystallin (**a**) and ovalbumin (**b**) in phosphate buffer were exposed to red and green light to different time intervals. The native signal is shown in black lines. Evident and consistent changes in protein secondary structure are observed after irradiation with red light. However, the effect of the green light is not so significant and regular with the doses in both proteins. See the text and Table 1 for details.

To gain insight about the secondary structure disturbance of the studied proteins, we analyzed the residues percentage in a given structural conformation. Figure 3 shows the CD spectrum of β_L_-crystallin (a) and ovalbumin (b) in a phosphate buffer after irradiation. It can be observed that red light on β_L_-crystallin gradually decreases the α-helix content and significantly increases the β-sheets, confirming the results obtained by DLS. In contrast, red light slightly increases the α-helix with no evident changes in beta-sheets for ovalbumin. According to our results, green light does not produce significant and consistent changes as the dose in both proteins is increased. The detailed secondary structure information of these spectra is presented in Table 1.

### Fluorescence emission spectra.

**Figure 4:**
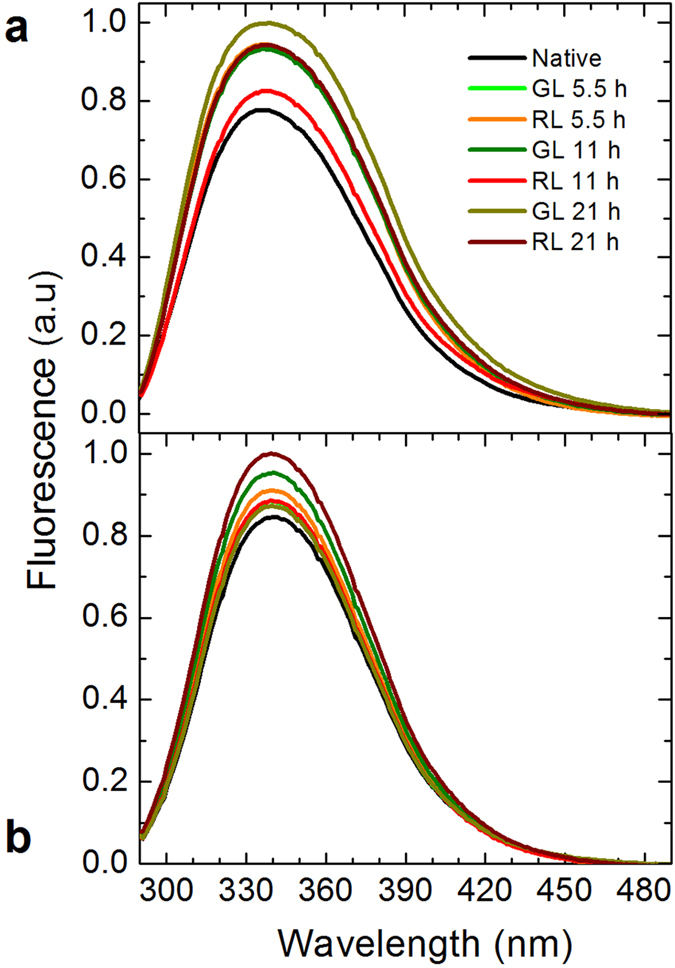
Fluorescence emission spectra. β_L_-crystallin (**a**) and ovalbumin (**b**) in phosphate buffer were exposed to red and green light to different time intervals. The intensity changes of the fluorescence are not due to heat generated by the radiation (the temperature during irradiations was 23 ± 2 °C). The black lines represent the signal of the native protein and the red and green lines the irradiated proteins. The protein concentration was 0.1 mg/mL in 10 mM phosphate buffer. The excitation wavelength was at 280 nm, and emission spectra were measured from 290–500 nm. The intensity of the fluorescence emission increases in both proteins, and there is not a shift in the maximum emission wavelength.

To further explore the change in the molecular properties of the irradiated proteins, we evaluated the interaction of aromatic residues with its microenvironment by fluorescence experiments (see Fig. 4). When tryptophan and tyrosine are excited, the native proteins show a maximum fluorescence emission at 333 and 334 nm, for β_L_-crystallin (a) and ovalbumin (b), respectively. After β_L_-crystallin is exposed to red and green light, the fluorescence intensities increase. These increments are due to conformation changes of the protein to a more compact structure, as confirmed by the DLS and calorimetry results. Indeed, it is well known that the environment affects the fluorescence intensity and wavelength of the maximum emission (λ_max_)^34^. When the solvent becomes more polar (for example, by the addition of water), λ_max_ shifts to the red and the intensity reduces; when the solvent turns into a less polar liquid, λ_max_ shifts to the blue and the intensity increases. Furthermore, the quantum yield of the fluorescence is more sensitive to the environment that λ_max_^34^. Since the protein shrinks by visible radiation, it is clear that the aromatic amino acids (more importantly, tryptophan) bury inside it, so the environment becomes less perturbed by the polar solvent (water), increasing therefore the intensity of the fluorescence (in other words, this effect is closely similar to the one described above: the intensity goes up because the protein suffers a structural modification as if a less polar solvent was added). Of course, this change in intensity is very minor (but measurable) so we cannot resolve a shift to shorter wavelengths for λ_max_. With respect to the second protein, ovalbumin, we observe the same effect but with less strong intensities.

## Discussion

In this study we investigated the effect of red and green light in β_L_-crystallin and ovalbumin. Our findings show that this radiation produces conformational modifications in both proteins. They get a bit more packed presumably due to an alteration of the hydrogen-bond network (see Figs 2 and 4). Since an additional thermal energy is needed to break new links, the calorimetric enthalpy increases (see Fig. 1). Simultaneously, the secondary structure composition of proteins is modified upon such radiation (see Fig. 3). These changes are evident in β_L_-crystallin and barely noticeable in ovalbumin. In the case of β_L_-crystallin, visible light promotes aggregation. Since according to our DLS experiments ovalbumin does not aggregate but the enthalpy does increase, it is evident that the cohesion responsible for the enthalpy increment is due to protein shrinkage. Our findings show that β_L_-crystallin and ovalbumin exposed to red and green light for periods of time greater than 5 hours give rise to conformational and thermodynamic changes. It is captivating to discover that alterations normally produced by X and UV radiation at very short times^35^ can be achieved with lower energies, especially red light. We believe our results may give clues for understanding some of the effects. Interestingly, β_L_-crystallin aggregates upon low energy radiation, which to the best of our knowledge is the first time reported. According to the data in the literature, 40 Jcm^−2^ of visible light is equivalent to outdoors midsummer sunlight exposure of approximately 15 min in Houston, TX^36^. This irradiance does not taken to account UV and IR. However, it is easy to estimate tha the maximum dose for green or red light in this study is equivalent to 10.2 Jcm^−2^. Then, around 20 mins under sunlight corresponds to the maximum dose in our experiments. It will be interesting to further investigate whether or not our findings correlate with the phenomenon of cataract formation in human eyes.

## Methods

### Proteins solution preparation

β_L_-crystallin from bovine eye lens (C5163 Sigma-Aldrich, USA) and albumin from chicken egg white (ovalbumin, A5253 Sigma-Aldrich, USA) were prepared at 25 °C. A stock solution of each protein was prepared as explained below. The concentration was verified by absorbance at 280 nm using a NanoDrop 2000 Spectrophotomer (Thermo Scientific, USA).

### Irradiation

Protein solutions were separately exposed to green (490–540 nm) and red (600–650 nm) light- emitting diodes (LEDs), LED-P3G-200/41 and LED-P3R-120/41, respectively. Four LEDs of the same wavelength were placed in an aluminium support and connected in parallel to a DC power supply (E3632A, Agilent) at 2.83 V for green and 2.05 V for red light. The intensity emitted was 4 × 10^3^ l× which was measured by a LX-1108 Lutron light meter. The samples were placed in a Petri dish on top (at a distance of 2.5 cm) of the LED arrangement, and irradiated for 5.5, 11 and 21 h (2.67, 5 and 10.2 J/cm^2^, respectively). The temperature during irradiations was 23 ± 2 °C which was monitored with a NI USB-TC01 thermocouple. Native and light-irradiated samples were obtained from the same stock. After irradiations, the samples were stored for 24 h at 4 °C before further analysis.

### Calorimetric analysis

Heat capacity profiles of the samples (native and irradiated) were recorded using a calorimeter (Microcalorimeter, NanoDSC, TA Instruments, USA) which was interfaced to a PC. Irradiated proteins were previously prepared by dissolving 2 mg of protein per milliliter of PBS buffer (0.01 M phosphate buffer, 0.0027 M potassium chloride and 0.137 M sodium chloride) with Milli- Q-water, at pH 7.2. Before the samples were loaded into the differential scanning calorimetry capillaries, they were degassed at 635 mmHg for 5 mins at 25 °C and 500 r.p.m. The β_L_-crystallin solution was heated from 30 to 80 °C and the ovalbumin 45 to 95 °C. The samples were measured at constant pressure (3 atm) and the heating rate was 1 °C/min. Each experiment was performed three times using different samples prepared under the same conditions. Data were analyzed using the software provided with the calorimeter.

### Dynamic light scattering

Particle size distribution was determined at 25 °C using a Zetasizer NanoZS (Malvern, UK). The protein suspensions of 1 mL (at a concentration of 0.1 mg/mL in phosphate buffer) were poured into disposable polystyrene cuvettes and exposed to a 633 nm He-Ne laser. Each measurement was performed two times, but each one is the average of twelve iterations.

### Circular dichroism

Circular dichroism (CD) spectroscopy was used to analyze the secondary structure of the proteins. CD spectra were recorded on a J-1500 spectropolarimeter (JASCO Inc., Easton MD, USA) using a 0.1 cm path length cell over the 190–240 nm range. The concentration of protein was 0.1 mg/mL in 10 mM phosphate buffer, pH 7.2, and the temperature regulated at 25 °C by a Peltier temperature-controlled cell holder (PTC-435, JASCO). CD spectra of the native and irradiated proteins were acquired every 0.1 nm with 1 s averaging time per point and a 1 nm band pass. Three measurements were made for each sample. Each spectrum was obtained as an average of three scans to reduce noise and smoothed before structure analysis was performed. The secondary structure prediction was performed using the CONTIN algorithm and SMP180 (optimised for 190–240 nm) reference sets, accessed through the Dichroweb server^37^.

### Fluorescence measurements

Intrinsic fluorescence of proteins was recorded on a Shimadzu RF-5301PC spectrofluorometer (Shimadzu Scientific Instruments, Columbia, MD, USA) equipped with a thermostatic cell chamber and constant stirring. The steady-state fluorescence emission spectra were recorded between 290–500 nm after exciting at 280 nm, 5 nm slit and at 25 °C. The protein concentration was 0.1 mg/mL in 10 mM phosphate buffer, pH 7.2.

## Additional Information

**How to cite this article**: Espinoza, J. Horacio. *et al.* Effects of green and red light in β_L_-crystallin and ovalbumin. *Sci. Rep.* **5**, 18120; doi: 10.1038/srep18120 (2015).

## Figures and Tables

**Table 1:** Secondary structure obtained from circular dichroism for β_L_-crystallin and ovalbumin exposed to different time intervals to red and green light.

**Native**	**α-helix(%)**	**β-sheet(%)**
	**β**_**L**_**-crystallin**	
	**10.4**	**38.0**
**Irradiated**		
** Green 5.5 h**	11.9	36.9
** Red 5.5 h**	11.7	37.8
** Green 11 h**	4.4	48.5
** Red 11 h**	2.7	49.4
** Green 21 h**	9.0	38.3
** Red 21 h**	2.3	51.1
**Native**	**Ovalbumin**	
	**20.2**	**30.0**
**Irradiated**		
** Green 5.5 h**	20.7	30.5
** Red 5.5 h**	18.6	33.0
** Green 11 h**	21.3	29.7
** Red 11 h**	19.6	32.9
** Green 21 h**	21.4	30.8
** Red 21 h**	22	30.5

The proportion of secondary structures was estimated from the deconvolution of CD spectra using the DICHROWEB online server with the CONTIN algorithm^37^.

## References

[b1] Sulkowsky E. *et al.* Inhibition of protein synthesis in yeast by low intensities of visible light. Nature 202, 36–39 (1964). 14166719 10.1038/202036a0

[b2] Kelly M. S. & Gay J. L. The action of visible radiation on the formation and properties of *Saccharomyces Ascospores*. Arch. Mikrobiol. 66, 259–272 (1969). 4194685 10.1007/BF00412058

[b3] Klein R. M. Effects of green light on biological systems. Biol. Rev. 67, 199–284 (1992). 1498206 10.1111/j.1469-185x.1992.tb01019.x

[b4] Folta K. M. & Maruhnich S. A. Green light: a signal to slow down or stop. J. Exp. Bot. 58, 3099–3111 (2007). 17630292 10.1093/jxb/erm130

[b5] Lee N. Y. *et al.* Effect of light emitting diode radiation on antioxidant activity of barley leaf. J. Korean Soc. Appl. Biol. Chem. 53, 685–690 (2010).

[b6] Olle M. & Virsile A. The effects of light-emitting diode lighting on greenhouse plant growth and quality. Agric. Food Sci. 22, 223–234 (2013).

[b7] Sohni J., Vojisavljevic V. & Pirogova E. Progress in Electromagnetics Research Symposium, PIERS Proceedings, Taipei, China, March 25-28, 2013; Curran Associates, Inc.: New York, , (2014).

[b8] Różanowska M. & Sarna T. Light-induced damage to the retina: role of rhodopsin chromophore revisited. Photochem. Photobiol. 81, 1305–1330 (2005). 16120006 10.1562/2004-11-13-IR-371

[b9] Korbee N., Figueroa F. L. & Aguilera J. Effect of light quality on the accumulation of photosynthetic pigments, proteins and mycosporine-like amino acids in the red alga *Porphyra leucosticta* (Bangiales, Rhodophyta). J. Photochem. Photobiol. B: Biol. 80, 71–78 (2005). 10.1016/j.jphotobiol.2005.03.002 16038805

[b10] Kuse Y. *et al.* Damage of photoreceptor-derived cells in culture induced by light emitting diode-derived blue light. Sci. Rep. 4, 1–12 (2014). 10.1038/srep05223 PMC4048889 24909301

[b11] Hori M. *et al.* Lethal effects of short-wavelength visible light on insects. Sci. Rep. 4, 1–6 (2014). 10.1038/srep07383 PMC4260232 25488603

[b12] Lee J.-B. *et al.* Blue light–induced oxidative stress in human corneal epithelial cells: protective effects of ethanol extracts of various medicinal plant mixtures, Invest. Ophthalmol. Vis. Sci. 55, 4119–4127 (2014). 24925877 10.1167/iovs.13-13441

[b13] Owaga K. *et al.* Protective effects of bilberry and lingonberry extracts against blue light-emitting diode light-induced retinal photoreceptor cell damage *in vitro*. BMC Complementary and Alternative Medicine 14, 120–130 (2014). 24690313 10.1186/1472-6882-14-120 PMC3992157

[b14] Klein R. M. & Edsall P. C. Interference by near ultraviolet and green light with growth of animal and plant cell cultures. Photochem. Photobiol. 6, 841–850 (1967). 6077701 10.1111/j.1751-1097.1967.tb08897.x

[b15] Organisciak D. T. & Vaughan D. K. Retinal light damage: mechanisms and protection Prog. Retin Eye Res. 29 113–134 (2010). 19951742 10.1016/j.preteyeres.2009.11.004 PMC2831109

[b16] Lin C. *et al.* Expression of an *Arabidopsis* cryptochrome gene in transgenic tobacco results in hypersensitivity to blue, UV-A, and green light. Proc. Natl. Acad. Sci. USA 92, 8423–8427 (1995). 7667306 10.1073/pnas.92.18.8423 PMC41169

[b17] Karu T. I. *et al.* Biostimulating action of low-intensity monochromatic visible light: is it possible? Laser Chem. 5, 19–25 (1984).

[b18] Idil O. *et al.* The effect of UV-A and various visible light wavelengths radiations on expression level of *Eschericia coli* oxidative enzymes in seawater *Jundishapur* J. Microbiol. 6, 230–236 (2013).

[b19] Muneer S. *et al.* Influence of green, red and blue light emitting diodes on multiprotein complex proteins and photosynthetic activity under different light intensities in lettuce leaves (*Lactuca sativa* L). Int. J. Mol. Sci. 15, 4657–4670 (2014). 24642884 10.3390/ijms15034657 PMC3975419

[b20] Schmid F. X. In Encyclopedia of Life Sciences, (MacMillan Publishers Ltd.) 1–4 (Nature Publishing Group, 2001).

[b21] Muranov K. O. *et al.* Mechanism of aggregation of UV-irradiated β_L_-crystallin. Exp. Eye Res. 92, 76–86 (2011). 21093434 10.1016/j.exer.2010.11.005

[b22] Chen J., Callis P. R. & King J. Mechanism of the very efficient quenching of tryptophan fluorescence in human γD- and γS-crystallins: the γ-crystallin fold may have evolved to protect tryptophan residues from ultraviolet photodamage. Biochemistry 48, 3708–3716 (2009). 19358562 10.1021/bi802177g PMC2674318

[b23] Mayr E., Jaenicke R. & Glockshuber R. Domain interactions and connecting peptides in lens crystallins J. Mol. Biol. 235, 84–88 (1994). 8289268 10.1016/s0022-2836(05)80017-8

[b24] Wang S., Leng X.-Y. & Yan Y.-B.. The benefits of being β-crystallin heteromers: βB1-crystallin protects βA3-crystallin against aggregation during co-refolding Biochemistry 50, 10451–10461 (2011). 22032798 10.1021/bi201375p

[b25] Bloemendal H. *et al.* Ageing and vision: structure, stability and function of lens crystallins Prog. Biophys. Mol. Biol. 86, 407–485 (2004). 15302206 10.1016/j.pbiomolbio.2003.11.012

[b26] Sharma K. K. & Santhoshkumar P. Lens agging: effects of crystallins. Biochim. Biophys. Acta 1790, 1095–1108 (2009). 19463898 10.1016/j.bbagen.2009.05.008 PMC2743770

[b27] Lampi K. J. *et al.* Deamidation in human lens βB2-crystallin destabilizes the dimer. Biochemistry 45, 3146–3153 (2006). 16519509 10.1021/bi052051k PMC2533042

[b28] Slingsby C. Structural variation in lens crystallins TIBS 10, 281–284 (1985).

[b29] Serebryany E. & King J. A. The βγ-crystallins: Native state stability and pathways to aggregation. Prog. Biophys. Mol. Biol. 115 32–41 (2014). 24835736 10.1016/j.pbiomolbio.2014.05.002 PMC4438767

[b30] Huntington J. A. & Stein P. E. Structure and properties of ovalbumin. J. Chromatography B 756, 189–198 (2001). 10.1016/s0378-4347(01)00108-6 11419711

[b31] Omana D. A. *et al.* Proteomic analysis of egg white proteins during storage. Proteomics 11, 144–153 (2011). 21182201 10.1002/pmic.201000168

[b32] Khanova H. A. *et al.* Mechanism of chaperone-like activity. Suppression of thermal aggregation of β_L_-crystallin by α-crystallin. Biochemistry, 44, 15480–15487 (2005). 16300396 10.1021/bi051175u

[b33] Shinohara H. *et al.* Transition of ovalbumin to thermostable structure entails conformational changes involving the reactive center loop. Biochem. Biophys. Acta 1770, 5–11 (2007). 16987608 10.1016/j.bbagen.2006.06.019

[b34] Stryer L. Fluorescence spectroscopy of proteins. Science 162, 526–533 (1968). 5706935 10.1126/science.162.3853.526

[b35] Donovan J. W. Changes in ultraviolet absorption produced by alteration of protein conformation. J. Biol. Chem. 244, 1961–1967 (1969). 4889460

[b36] Liebel F., *et al.* Irradiation of skin with visible light induces reactive oxygen species and matrix-degrading enzymes. J. Invest Dermatol, 132, 1901–1907 (2012). 22318388 10.1038/jid.2011.476

[b37] Whitmore L. & Wallace B. A. DICHROWEB, an Online server for protein secondary structure analyses from circular dichroism spectroscopic data. Nucleic Acids Res. 32, W668–W673 (2004). 15215473 10.1093/nar/gkh371 PMC441509

